# Simulation Study of Intermittent Axonal Block and Desynchronization Effect Induced by High-Frequency Stimulation of Electrical Pulses

**DOI:** 10.3389/fnins.2018.00858

**Published:** 2018-11-22

**Authors:** Zheshan Guo, Zhouyan Feng, Yang Wang, Xuefeng Wei

**Affiliations:** ^1^Key Lab of Biomedical Engineering for Ministry of Education, College of Biomedical Engineering and Instrument Science, Zhejiang University, Hangzhou, China; ^2^Department of Biomedical Engineering, The College of New Jersey, Ewing, NJ, United States

**Keywords:** high-frequency stimulation, potassium accumulation, axonal block, desynchronization, model of myelinated axon

## Abstract

Deep brain stimulation (DBS) has been successfully used in treating neural disorders in brain, such as Parkinson’s disease and epilepsy. However, the precise mechanisms of DBS remain unclear. Regular DBS therapy utilizes high-frequency stimulation (HFS) of electrical pulses. Among all of neuronal elements, axons are mostly inclined to be activated by electrical pulses. Therefore, the response of axons may play an important role in DBS treatment. To study the axonal responses during HFS, we developed a computational model of myelinated axon to simulate sequences of action potentials generated in single and multiple axons (an axon bundle) by stimulations. The stimulations are applied extracellularly by a point source of current pulses with a frequency of 50–200 Hz. Additionally, our model takes into account the accumulation of potassium ions in the peri-axonal spaces. Results show that the increase of extracellular potassium generates intermittent depolarization block in the axons during HFS. Under the state of alternate block and recovery, axons fire action potentials at a rate far lower than the frequency of stimulation pulses. In addition, the degree of axonal block is highly related to the distance between the axons and the stimulation point. The differences in the degree of block for individual axons in a bundle result in desynchronized firing among the axons. Stimulations with higher frequency and/or greater intensity can induce axonal block faster and increase the desynchronization effect on axonal firing. Presumably, the desynchronized axonal activity induced by HFS could generate asynchronous activity in the population of target neurons downstream thereby suppressing over-synchronized firing of neurons in pathological conditions. The desynchronization effect generated by intermittent activation of axons may be crucial for DBS therapy. The present study provides new insights into the mechanisms of DBS, which is significant for advancing the application of DBS.

## Introduction

Deep brain stimulation (DBS) is an effective clinical treatment for diseases of motor nervous system, such as Parkinson’s disease, essential tremors, and dystonia (Mehanna and Lai, 2013; Cury et al., 2017). It also exhibits potentials in treating epilepsy and other mental illness such as depression and obsession (Bergey, 2013; Blomstedt et al., 2013; Narang et al., 2016). However, the precise mechanisms of DBS action are still under debate.

Normally, DBS therapy is performed with continuous stimulation of electrical pulses. The efficacy of DBS is strongly related to the frequency of pulses, which is adjusted for optimal outcome of individual patient. The frequency range of effective stimulation in clinic is 90–200 Hz (commonly around 130 Hz), hence the stimulation is called high-frequency stimulation (HFS) (Birdno and Grill, 2008; Eusebio et al., 2011; Zhong et al., 2011; McConnell et al., 2012).

Both HFS and lesion therapy are found to produce similar effects on the relief of symptoms, thus HFS is originally assumed to inhibit the neuronal activity of stimulated areas in brain (Boraud et al., 1996; Anderson, 2006). For example, HFS activates axon terminals connecting inhibitory synapses, thus enhances the release of inhibitory neurotransmitters thereby inhibiting the activity of postsynaptic neurons (Johnson and McIntyre, 2008; Deniau et al., 2010; Chiken and Nambu, 2013). However, some research showed a contrary effect that HFS could facilitate the action potential firing of target neurons (Reese et al., 2011; Cleary et al., 2013). A recent hypothesis claims that desynchronization is more noteworthy than excitability change (Medeiros Dde and Moraes, 2014). Since over-synchronized activity of neurons is a pathological feature for many brain disorders such as Parkinson’s disease and epilepsy (Hammond et al., 2007; Jiruska et al., 2013), the role of HFS might be to decrease the synchronization among neurons (Deniau et al., 2010; Medeiros Dde and Moraes, 2014; Feng et al., 2017). However, it is not clear how HFS generates desynchronization.

Electrical pulses delivered from stimulating electrode are applied simultaneously on different elements of surrounding neurons, among which axon membrane is most inclined to be activated. Action potential may initiate at axon, even if soma locates closer to the stimulating electrode (Ranck, 1975; Nowak and Bullier, 1998; McIntyre and Grill, 1999). Therefore, the response of axon may play an important role in the action of DBS (Chomiak and Hu, 2007; Udupa and Chen, 2015). Some research showed that continuous HFS could generate depolarization block on axons, making axons fail to fire an action potential following every stimulating pulse (Jensen and Durand, 2009; Zheng et al., 2011; Feng et al., 2013, 2014). This depolarization block of axons may be an important mechanism underlying the desynchronization effect of HFS.

Previous studies suggest that accumulation of K^+^ in peri-axonal sub-myelin space during HFS contributes to the depolarization block of axons (Bellinger et al., 2008; Zheng et al., 2011). We hypothesize here that the depolarization block could be intermittent because of the fluctuation of sub-myelin K^+^ concentration during HFS thereby causing the axons to fire action potentials at a rate far lower than the frequency of stimulation pulses. Under this situation, the axonal firing generated within an axon bundle would be asynchronous thereby desynchronizing neuronal activity in the downstream projecting area.

To verify this hypothesis, we developed a computational model to simulate thin myelinated axons in brain and to study the effects of HFS on single and multiple axons (an axon bundle). Since current techniques of *in vivo* experiments do not allow intracellular recordings of multiple thin axons simultaneously to trace their reactions, this modeling study is significant for unraveling the desynchronization mechanism of axonal role in DBS.

## Materials and Methods

To investigate the responses of axons to HFS, we utilized a computational model of thin myelinated axon in the central nervous system using NEURON v7.4. The axonal model was modified from a previous model (Bellinger et al., 2008). Bellinger’s model was adapted from motor nerve fibers (McIntyre and Grill, 2002; McIntyre et al., 2004). To simulate more closely the responses of brain neurons to HFS, we replaced the kinetic equations of ionic channels with those from axons of pyramidal neurons of brain (Bianchi et al., 2012) and altered other parameters such as axonal diameters, lengths of internode parts, and number of myelin lamella accordingly. Details of the model are described below, including the structures and parameter settings, the accumulation of extracellular K^+^, the administration of stimulation by a point source of current pulses extracellularly, as well as signal recording and analyzing.

### Morphological Parameters of the Axon Model

**FIGURE 1:**
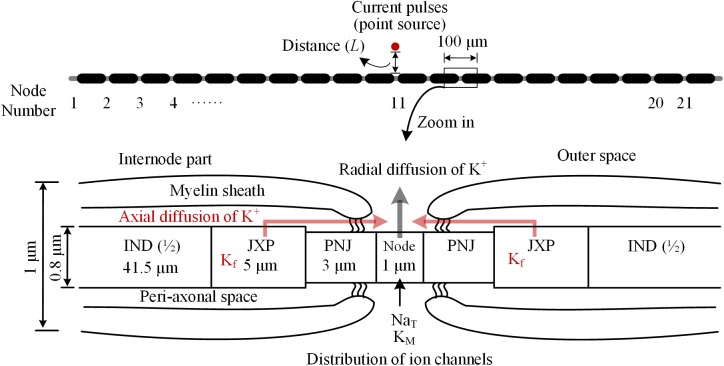
Schematic diagram of the model for thin myelinated axon, including structures, dimensions, and distributions of ionic channels. *Top*: Overview of the entire axon with 21 nodes of Ranvier (Node) and 20 internode parts. *Bottom*: Magnified view of a nodal region with the three sections surrounding each node: paranodal junction (PNJ), juxtaparanodes (JXP), and internode (IND). Axial and radial diffusion of K^+^ are shown by pink and gray arrow-lines, respectively.

The myelinated axon consists of 21 nodes of Ranvier (abbreviated as Node) and 20 internode parts (Figure 1). The outer diameter of the myelin sheath is set to 1 μm to simulate the thin axons of brain neurons (Wang et al., 2008). Thus, the internode length is set to 100 μm according to the ratio of axon diameter to internode length of 1:100 (Frijns et al., 1994). In this case, the total length of the axon is ∼2 mm. Additionally, according to the linear relationship between the thickness of myelin sheath and axon diameter (Schröder, 1972), the number of myelin lamellae is set to 15 layers. The morphological parameters of different parts of the axon model are listed in Table 1.

**Table 1:** Morphological parameters of the axon model.

Parameter	Value	Unit
Outer diameter of axon	1	μm
Internode length	100	μm
Total axon length	2	mm
Node diameter	0.7	μm
Node length	1	μm
PNJ diameter	0.7	μm
PNJ length	3	μm
JXP diameter	0.8	μm
JXP length	5	μm
IND diameter	0.8	μm
IND length	83	μm
PNJ peri-axonal space width	1.8	nm
JXP peri-axonal space width	8	nm
IND peri-axonal space width	8	nm
Number of myelin lamella	15	

As shown in Figure 1, inside the myelin sheath, each internode part is divided into two halves, each with three sections: a paranodal junction (PNJ), a juxtaparanode (JXP), and ½ internode (IND), from either side to the middle (Bellinger et al., 2008; Rasband and Peles, 2016). Therefore, each internode part contains two PNJ, two JXP, and one IND. These sections differ in both length and diameter. Particularly, IND is far longer than PNJ and JXP. “Peri-axonal space” refers to the space between axon membrane and surrounding myelin sheath, and its width changes along the internode parts (see Table 1).

### Electrical Parameters of the Axon Model

The axon contains both passive and active electrical features (Table 2). The parameters of passive features include: resistivity for the axoplasm (ρ_a_); capacitance (c_m_), leakage conductance (g_Lk_), and leakage reversal potential (E_Lk_) for the axon membrane; capacitance (c_my_) and conductance (g_my_) for the myelin. Resting potential of the axon membrane is set to -66 mV (Staff et al., 2000). The sodium (Na^+^) reversal potential is set to a fixed value 45 mV (Bianchi et al., 2012), whereas the potassium (K^+^) reversal potential varies with the changes of extracellular K^+^ concentration during HFS. The initial value of K^+^ reversal potential is -95 mV calculated by Nernst equation based on the initial K^+^ concentrations inside and outside the axon membrane (Table 3).

**Table 2:** Electrical parameters of the axon model.

Parameter	Value	Unit
Axoplasmic resistivity (ρ_a_)	70	Ω⋅cm
Capacitance of the axon membrane (c_m_)	1	μF/cm^2^
Leakage conductance (g_Lk_)	0.0001	S/cm^2^
Leakage reversal potential (E_Lk_)	-66	mV
Capacitance of the myelin (c_my_)	0.1	μF/cm^2^
Conductance of the myelin (g_my_)	0.001	S/cm^2^
Resting potential	-66	mV
Potassium reversal potential (initial value)	-95	mV
Sodium reversal potential	45	mV
Max. conductance of transient Na^+^ (Na_T_) on Node	2	S/cm^2^
Max. conductance of M-type K^+^ (K_m_) on Node	0.003	S/cm^2^
Max. conductance of fast K^+^ (K_f_) on JXP	0.03	S/cm^2^

**Table 3:** Parameters for modeling dynamics of K^+^ concentration.

Parameter	Value	Unit
Intracellular K^+^ concentration	106	mM
Extracellular K^+^ concentration (initial value)	3	mM
Intracellular Na^+^ concentration	10	mM
Diffusion coefficient (*D*)	1.85	μm^2^/ms
Axial area of diffusion zone at IND	0.02	μm^2^
Axial area of diffusion zone at JXP	0.02	μm^2^
Axial area of diffusion zone at PNJ	0.004	μm^2^
Radial area of diffusion zone at Node	2.2	μm^2^
Max. current of NaK pump (I_NaKmax_)	2.46	μA/cm^2^
K^+^ equilibrium binding constant (KmK)	5.3	mM
Na^+^ equilibrium binding constant (KmNa)	27.9	mM

The parameters of active features include the values of maximum conductance for three types of voltage-gated ionic channels: transient Na^+^ channel (Na_T_) and M-type K^+^ channel (K_M_) distributed on Node membrane; fast K^+^ channel (K_f_) distributed on JXP membrane (Table 2 and Figure 1).

At node membrane, the Na_T_ channels mainly correspond to an integration of sodium channel isoforms of Nav1.1 and Nav1.6 (Caldwell et al., 2000; Duflocq et al., 2008). The kinetic equation of Na_T_ channels is adopted from literature (Bianchi et al., 2012), which was modified from the work with experimental results (Migliore et al., 1999). The maximum conductance of Na_T_ is set to 2 S/cm^2^ according to the range of maximum Na^+^ conductance 1.3 ∼ 2.6 S/cm^2^, i.e., 1000 ∼ 2000 channels/μm^2^ and 13 pS/channel (Scholz et al., 1993; Waxman and Ritchie, 1993). The K_M_ channels represent KCNQ isoforms (Kv7.2/7.3) (Devaux, 2004; Schwarz et al., 2006). The kinetic equation and maximum conductance of K_M_ are adopted from literature (Bianchi et al., 2012). At JXP membrane, the K_f_ channel has been commonly used in computation modeling (Schwarz et al., 1995; McIntyre et al., 2004; Bellinger et al., 2008) to represent the ionic current of potassium corresponding to an integration of Kv isoforms such as Kv1.1 and Kv1.2 (Wang et al., 1993; Rasband and Trimmer, 2001; Rasband and Peles, 2016). The kinetic equation of K_f_ is adopted from previous models (McIntyre et al., 2004), and its maximum conductance is set to 0.03 S/cm^2^ (Bellinger et al., 2008). The three types of voltage-gated channels are dominant for generation and conduction of action potentials in myelinated brain axons (Rasband and Peles, 2016; Nelson and Jenkins, 2017).

### Change of K^+^ Concentrations in the Peri-Axonal Space

Our axon model also takes into account NaK pumps and K^+^ diffusion as previous report (Bellinger et al., 2008). The concentration of K^+^ inside the axon membrane is set to constant 106 mM, and K^+^ concentration outside of the axon membrane ([K^+^]_o_) is initially set to 3 mM (Table 3). The K^+^ inside the membrane can only outflow through the K^+^ channels on the Node and JXP membranes. The outflowing K^+^ on the Node diffuses to the outer space radially. The outflowing K^+^ on the JXP can first flow into the peri-axon space, then diffuse axially to the outside of the Node and finally diffuse into outer space radially (Figure 1).

Both axial and radial diffusions of K^+^ follow the Fick’s law:

(1) $$J=D\times A\times \left(\frac{\text{d}{\left[{\text{K}}^{\text{+}}\right]}_{\text{o}}}{\text{d}x}\right)$$

where *J* is the diffusion flux; *D* is the diffusion coefficient 1.85 μm^2^/ms; *A* is the cross section area of the diffusion zone; [K^+^]_o_ is extracellular K^+^ concentration; *x* is the diffusion distance. Based on the data in Table 1, the axial area of the peri-axon space outside IND, JXP, and PNJ are 0.02, 0.02, and 0.004 μm^2^, respectively; the radial area of Node surface is 2.2 μm^2^, much larger than the axial area of peri-axon space (Table 3).

[K^+^]_o_ is also regulated by NaK pump, which pumps out three Na^+^ and pumps in two K^+^ per ATP hydrolyzed. Assuming the NaK pumps are distributed evenly on the entire axon membrane, the ionic current (*I*_NaK_) of NaK pump per unit area is (Bellinger et al., 2008):

(2) $$I_{\text{NaK}}={\text{I}}_{\text{NaKmax}}\left(\frac{{\left[{\text{K}}^{\text{+}}\right]}_{\text{o}}}{{\left[{\text{K}}^{\text{+}}\right]}_{\text{o}}+\text{KmK}}\right)\left(\frac{{\left[{\text{Na}}^{\text{+}}\right]}_{\text{i}}^{1.5}}{{\left[{\text{Na}}^{\text{+}}\right]}_{\text{i}}^{1.5}+{\text{KmNa}}^{1.5}}\right)\times \left(\frac{V+150}{V+200}\right)$$

where *V* is the membrane potential; [Na^+^]_i_ is intracellular Na^+^ concentration; I_NaKmax_ is the maximum transport current per unit area; KmK and KmNa are the equilibrium binding constants of K^+^ and Na^+^, respectively (Table 3).

The parameter values of formulae (1) and (2) are listed in Table 3. The two formulae were used to simulate the dynamic changes of the K^+^ concentrations outside axon membrane.

### Extracellular Stimulation and Signal Recording

Electrical stimulation is a sequence of monophasic current pulses with pulse width of 0.1 ms, pulse intensity of -0.1 to -0.5 mA, pulse frequency of 50, 130, or 200 Hz, and stimulation duration of 1 min.

Extracellular point source of stimulation is located above the 11th node of Ranvier (Node_11_), which is the center of axon (Figure 1). The extracellular potential (*φ*) at a specific part on the axon membrane generated by the stimulation point is:

(3) φ=I/4πσr

where *σ* is the extracellular conductivity, which is set to 0.286 S/m; *I* is the pulse intensity of stimulations; *r* is the distance between the stimulation point, and the specific part of the axon.

An effective action potential induced by stimulation is defined as one that can spread to both axon ends successfully. Because the action potentials induced at the central node (Node_11_) can spread in both orthodromic and antidromic directions equally, the numbers of effective action potentials during stimulation periods are counted only at one end node (Node_1_) to evaluate the axonal conduction. Also, the membrane potential of the central Node_11_ together with the [K^+^]_o_ in the peri-axonal space of the neighboring JXP are recorded to investigate the relation between accumulated K^+^ and HFS-induced action potentials.

Additionally, an index called “induction ratio” of axonal action potentials was used to evaluate the degree of intermittent depolarization block induced by the stimulations. The value of the index, represented as percentage, was defined as the reciprocal of the number of stimulation pulses in between two successive evoked action potentials. For example, if a second action potential is evoked following three successive pulses, that is, two of the pulses fail to induce effective action potential; then the induction ratio is 33%.

### Evaluation of Action Potential Synchronization by Responses of an Axon Bundle to Stimulation

To study the integrate responses of multiple axons to HFS, 11 identical axons are spaced in parallel at an interval of 10 μm in a simplified model of axon bundle. The stimulation point is located at the same plane as the axons, above the axon center (Node_11_) of the topmost axon with a distance of 20 μm. Thus, the distances (*L*) from the stimulation point to the central nodes (Node_11_) of the 11 axons are in a range of 20–120 μm. Percentage ratio of effective action potentials in the 11 axons induced by each pulse is calculated, which is defined as “synchronization ratio.” If all of the 11 axons are activated by a pulse synchronously, the synchronization ratio is 100%. The curve of synchronization ratio is smoothed by sliding average of every 10 data points.

## Results

### [K^+^]_o_ Fluctuation Induces Intermittent Axonal Block

**FIGURE 2:**
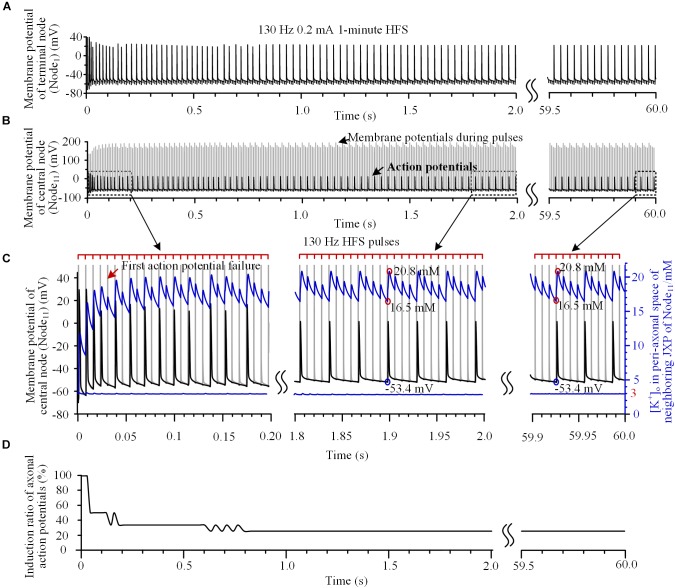
Action potentials and [K^+^]_o_ induced by high-frequency stimulation (HFS). **(A**,**B)** Membrane potentials recorded simultaneously at the end node (Node_1_) and the central node (Node_11_) during a 1-min train of 130 Hz pulse stimulation with 0.2 mA intensity. For clarity, recordings in 2.0 ∼ 59.5 s are omitted. **(C)** Magnified plots of membrane potentials at Node_11_ (black curves), [K^+^]_o_ at Node_11_ (blue curves below) and [K^+^]_o_ in the peri-axonal space of neighboring JXP (blue curves up) during the first 0.2 s, middle 1.8 ∼ 2.0 s and last 0.1 s of the 1-min HFS. Cathodic monophasic pulses of extracellular HFS are represented by the tick marks on the red lines above. The gray curves in **(B,C)** represent changes of membrane potentials induced by pulses at Node_11_. These large potentials are trimmed in the enlarged plots in **(C)**. **(D)** Change of induction ratio of axonal action potentials by pulses of HFS.

Our simulation results showed dynamics of the axonal responses to the pulses of HFS. For example, when a 130 Hz HFS was applied 50 μm above the central node (Node_11_) of axon (Figures 2A,B), the initial four pulses could all induce action potentials, which propagated to the end of axon (Node_1_) (Figures 2A,C). However, the fifth pulse only caused a change of potential on the Node_11_ membrane during the pulse and did not induce an action potential following the pulse (see the red arrow in Figure 2C). During the HFS, the induction ratio of action potentials gradually decreased from 100% to a steady value of 25% after 1.5 s of stimulation and maintained to the end of 1-min stimulation (Figure 2D), indicating that only one of every four pulses induces an action potential, intermittently.

To test the hypothesis that the intermittent block of axon results from the fluctuation of sub-myelin [K^+^]_o_ during HFS, we monitored [K^+^]_o_ within the peri-axon space of JXP next to the central node (Node_11_). During the initial four pulses of HFS, the [K^+^]_o_ increased rapidly from baseline 3 mM to over 14 mM (see the blue curve and right coordinate in Figure 2C). After the induction ratio of action potentials became stable (∼1.5 s after the onset of HFS), [K^+^]_o_ fluctuated with lower and upper limits of 16.5 and 20.8 mM, respectively. The outflow of K^+^ during each action potential caused a large jump in [K^+^]_o_ between the two concentration limits (circled in red on the blue curve in Figure 2C). A pulse that did not induce an action potential also caused a small increase in [K^+^]_o_ due to an elevation of membrane potential. Because of the persistent effects of NaK pump and ion diffusion that remove K^+^ from the peri-axon space, [K^+^]_o_ gradually decreased in a saw-toothed fashion in the intervals of action potentials and in the intervals of pulses. The changes of [K^+^]_o_ were also steady after ∼1.5 s of stimulation accompanying the stabilization of induction ratio of action potentials. Since the axonal activity changed from transient to steady state after ∼1.5 s of stimulation, we only provide the data of first 2-s stimulation in the subsequent results.

The increase of [K^+^]_o_ elevated the basic membrane potential of Node_11_ from -66 mV up to above -53.4 mV. When the [K^+^]_o_ intermittently fell below 16.5 mM and the membrane potential fell below -53.4 mV, an action potential could be induced again (circled by blue on the black curve in Figure 2C). Nevertheless, the [K^+^]_o_ immediately outside Node_11_ stayed at ∼3 mM during the entire HFS (Figure 2C, the blue line below). Therefore, the membrane depolarization caused by the increase of [K^+^]_o_ within the peri-axon space of JXP might be the reason why the axon failed to fire an action potential following each stimulation pulse.

**FIGURE 3:**
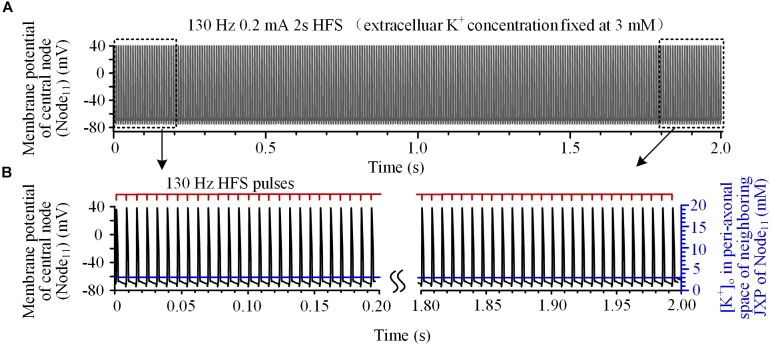
Axonal responses during high-frequency stimulation (HFS) when the extracellular K^+^ concentration was fixed at 3 mM: **(A)** Action potentials induced by 130 Hz pulses during 2-s HFS. **(B)** Membrane potential at Node_11_ (black curves) and [K^+^]_o_ in the peri-axonal space of neighboring JXP (blue curves) during the first and last 0.2 s of the 2-s HFS shown in **(A)**. Every stimulation pulse induces an action potential.

To verify this hypothesis, the mechanism of K^+^ accumulation outside the axon membrane was removed, i.e., the [K^+^]_o_ was fixed at 3 mM. Then, during the same 130 Hz stimulation, every pulse could induce an action potential at Node_11_ (Figures 3A,B) that spread to the axon ends.

These results indicate that the fluctuation of [K^+^]_o_ induced by HFS causes intermittent block and intermittent recovery in axons, which results in a firing rate of axonal action potentials far lower than the pulse frequency of HFS. For multiple axons, their action potentials might be induced by different pulses thereby resulting in misalignment in time and asynchronous firing of action potentials. We next test the hypothesis by simulating the responses of multiple axons to HFS.

### Asynchronous Firing of Multiple Axons Induced by High Frequency Stimulation

**FIGURE 4:**
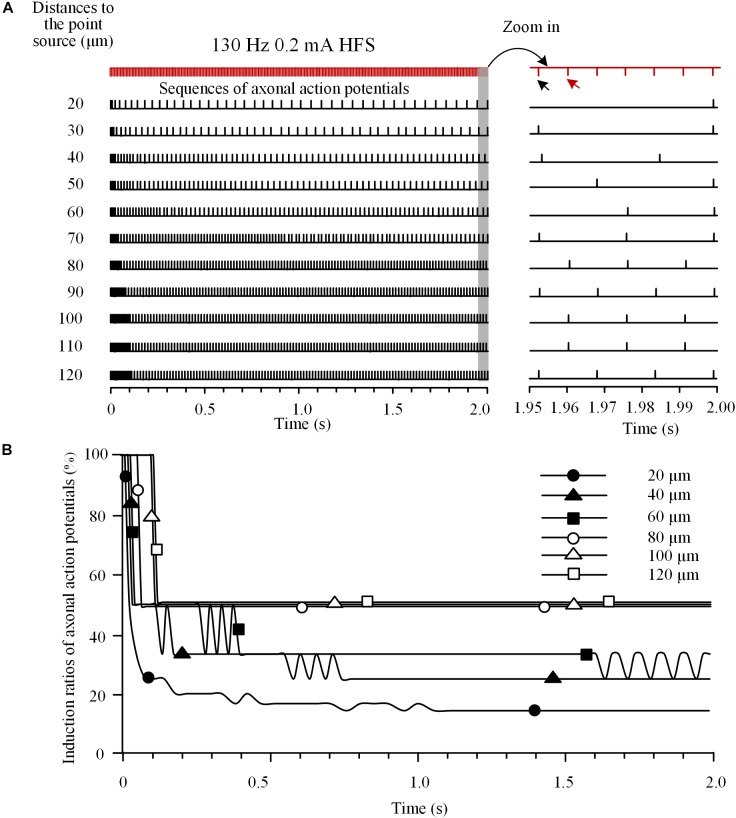
Responses of a bundle of axons to high-frequency stimulation (HFS). **(A)** Sequences of action potentials generated by 130 Hz HFS (0.2 mA) in 11 axons with different distances to the point source of stimulation. Axonal responses to the last seven pulses at the end of 2-s HFS are expanded at *right*. **(B)** Changes of induction ratios of action potentials for the six axons with distances of 20, 40, 60, 80, 100, and 120 μm to the stimulation source during HFS.

To investigate the integrated activity induced on multiple axons located at various distances from a stimulation point, we analyzed the responses of an axon bundle composed of 11 axons to HFS. Due to the different distances of axons to the stimulation point, the degree of axonal block was different for individual axons (Figure 4A). Axons closer to the stimulation point had lower induction ratios of effective action potentials. During a HFS train of 130 Hz with 0.2 mA intensity, after ∼1.5 s stimulation, the steady-state values of induction ratio were ∼14% for the axon with the shortest distance of 20 μm and ∼50% for the axon with the longest distance of 120 μm (Figure 4B).

The differences of block degrees would cause differences in firing time of the multiple axons responding to stimulation pulses thereby resulting in asynchronous firing of action potentials during HFS. For example, during the last seven pulses of HFS period (Figure 4A *right*), the first of the seven pulses (indicated by the black arrow) induced action potentials only on axons at 30, 40, 70, 90, and 120 μm distances, while the next pulse (indicated by the red arrow) induced action potentials only on axons at 80, 100, and 110 μm. Therefore, the two pulses induced asynchronous action potentials in different axons.

**FIGURE 5:**
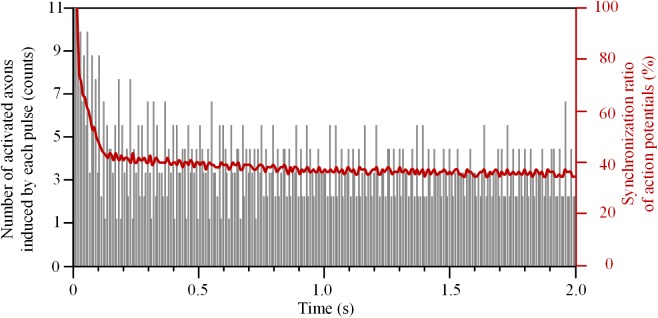
Synchronization ratio of action potentials generated in a bundle of 11 axons during high-frequency stimulation.

The number of axons activated by each pulse decreased rapidly at the beginning of HFS (Figure 5). The initial pulses could induce action potentials simultaneously in all 11 axons, and thereby the synchronization ratio of action potentials was 100%. After that, the synchronization ratio decreased and was down to a steady value of ∼36% after 1.5 s stimulation.

These results indicate that HFS could cause various degrees of depolarization block to the axons within a bundle, which decreased the synchronization of the overall firing of action potentials. However, the activation of axons was related to not only the distance from the stimulation point, but also the frequency and intensity of the stimulation. Therefore, we next examined the changes of synchronization ratios of multiple axons by stimulations of various frequencies and intensities.

### Effect of Stimulation Frequency and Intensity on the Synchronization of Induced Action Potentials in Axon Bundle

**FIGURE 6:**
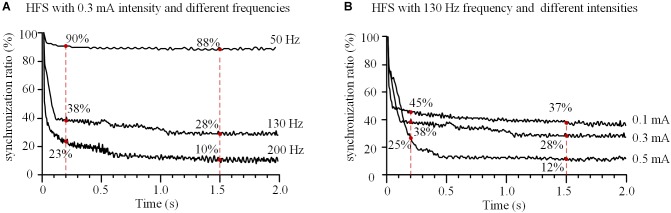
Effects of frequency and intensity of high-frequency stimulation (HFS) on the synchronization of action potential firing of an axonal bundle. **(A)** Changes of the synchronization ratios during HFS with different frequencies at a fixed intensity (0.3 mA). **(B)** Changes of the synchronization ratios during HFS with different intensities at a fixed frequency (130 Hz).

With a fixed stimulation intensity of 0.3 mA, HFS at different frequencies of 50, 130, and 200 Hz decreased the synchronization ratio of the 11 axons with different speeds (Figure 6A). For a lower frequency of 50 Hz, the synchronization ratio decreased slowly to ∼90% at 0.2 s and stabilized at ∼88% after 1.5 s. For higher frequencies of 130 and 200 Hz, the synchronization ratios decreased more rapidly, to ∼38 and ∼23% at 0.2 s, and stabilized at ∼28 and ∼10% after 1.5 s, respectively. These results indicate that the higher the HFS frequency, the faster the actions of axons desynchronized and the lower synchronization ratios at steady state.

With a fixed stimulation frequency of 130 Hz, HFS at different stimulation intensities of 0.1, 0.3, and 0.5 mA decreased the synchronization ratio of the axons in the assumed bundle to different degrees (Figure 6B). At 0.2 s, the synchronization ratios decreased to ∼45, ∼38, and ∼25%, respectively; after 1.5 s, they stabilized at ∼37, ∼28, and ∼12%, respectively, for the three stimulation intensities. These results indicate that the greater the stimulation intensity, the more desynchronized the action potentials of axons and the deeper the degree of axonal block.

## Discussion

In the present study, we utilized a computational model to investigate the responses of thin myelinated axons in brain to high-frequency pulse stimulation (HFS). The major findings are (1) the accumulation of K^+^ in the peri-axon space during HFS can cause intermittent depolarization block in axons and result in a firing rate of action potentials in axons far lower than the stimulation frequency; (2) the HFS-induced intermittent block can generate asynchronous firing of action potentials in multiple axons within a bundle; (3) differences in frequency and intensity of HFS generate different degrees of the axonal block and of the asynchronous activity thereby resulting in different modulation effects of HFS. Possible mechanisms of these findings and their implications are discussed below.

### Intermittent Depolarization Block and Its Implications

*In-vitro* studies on central nerves have shown that repeated stimulation can elevate [K^+^]_o_ thereby resulting in a decreased speed of axonal conduction until final block of the conduction. Artificially increasing [K^+^]_o_ can also generate axonal block (Förstl et al., 1982; Poolos et al., 1987). The simulation results of our study are consistent with those previous studies. Nevertheless, we further reveal an intermittent feature of the axonal block.

The stimulation-induced depolarization and action potentials are generated mainly in the Ranvier nodes, not in the internode parts, because of the high impedance of myelinated membrane of internodes and the sole distribution region of Na^+^ channel in the nodes (Figure 1). In contrast, the K^+^ accumulation is generated in the internode parts (Figure 2C), not immediately outside the nodes, because of the small peri-axon space around axon membrane of internodes and the dense distribution of K^+^ channel in the JXP section (Debanne et al., 2011). Due to a fast diffusion of K^+^ outside the Ranvier nodes (imitating the K^+^ buffering effect of nearby glial cells), our simulation results show that the [K^+^]_o_ at nodes stays at ∼3 mM (Figure 2C), and does not change obviously. Nevertheless, the accumulated [K^+^]_o_ around JXP sections and the excitation of nearby nodes can still interact with each other by the spread of potential changes along axonal membrane.

With the interaction, the HFS-induced depolarization of nodes can increase [K^+^]_o_ of JXP repeatedly following each stimulation pulse; whereas the increased [K^+^]_o_ in turn can cause persistent depolarization of axonal membrane by decreasing the Nernst potential of K^+^. A substantial depolarization may lead to depolarization block via the inactivation of sodium channels on the node and prevent the node from continuously generating action potential following each pulse of HFS (Hille, 2001; Qian et al., 2014; Kameneva et al., 2016). K^+^ accumulation in JXP sections is counteracted by K^+^ clearance via mechanisms of NaK pump and K^+^ diffusion. An action potential can be re-induced by a stimulation pulse when [K^+^]_o_ returns to an adequately low level (Figure 2C), which generates intermittent block and recovery of axonal activity.

Previous experimental studies have shown that HFS can induce partial block of axonal conduction, but not complete (Jensen and Durand, 2009; Zheng et al., 2011). Based on extracellular recording of population spikes from multiple axons under HFS, two possible mechanisms might underlie the partial block: (1) a part of the axons are completely blocked and the rest are not; (2) each axon produces intermittent block. Previous simulation study has suggested that the mechanism for axonal depolarization block is the former (Bellinger et al., 2008). If it was true that a part of the axons were completely blocked, the downstream projecting neurons would respond in two opposite scenario: either remain silent, or fire at a frequency close to the stimulation. However, the results of *in vivo* animal experiments suggest otherwise; the firing frequency of downstream neurons increase during HFS, but much lower than the stimulation frequency (Feng et al., 2017). Moreover, completely blocking of axon fibers requires stimulation frequencies up to thousands of Hertz (Kilgore and Bhadra, 2014; Couto and Grill, 2016), and the frequencies below 200 Hz used in clinical DBS are unlikely to produce complete axonal block. Therefore, the intermittent axonal block mechanism presented in this paper provides a more reasonable explanation for the experimental observations.

### Asynchronous Neuronal Firing and Its Implications

Our simulation results on an axon bundle indicate that intermittent block of individual axons can cause asynchronous firing of the entire axon bundle (Figures 4, 5). Axons at various distances from the stimulation point experience various degrees of depolarization block. Upon intermittent recovery from the block, these axons fire action potentials at different rates and different timings thereby generating asynchronous firing.

HFS-induced desynchronization has important implication to the mechanisms of DBS therapy, because synchronized firing is related to pathological conditions of many brain disorders. In movement disorders such as Parkinson’s disease, there is an increase in synchronization of neuronal activity in the basal ganglia and thalamus (Birdno and Grill, 2008; Gale et al., 2008). In epilepsy, populations of neurons fire excessively and synchronously at the onset of seizures (Lopes da Silva et al., 2003; Shiri et al., 2016). HFS could induce asynchronous firing of target neurons to replace the pathological synchronous activity in Parkinson’s disease (Cleary et al., 2013; Udupa and Chen, 2015), or suppress hyper-synchronous epileptiform activity (Good et al., 2009; Medeiros Dde and Moraes, 2014). Previous experimental study has proposed an intermittent block of axonal excitation as a possible mechanism for the generation of desynchronization by HFS (Feng et al., 2017). However, the underling mechanism of intermittent block has not been determined.

To our knowledge, this is the first simulation study addressing the intermittent block of axons and the desynchronization effect induced by HFS by incorporating the mechanism of submyelin K^+^ accumulation from the scale of axonal ultrastructure. Although the effect of extracellular K^+^ accumulation on axonal block was previously simulated in a modeling study (Bellinger et al., 2008), the simulation only presented a complete block of axon, not intermittent block. Therefore, it cannot explain the desynchronization mechanism of HFS.

Moreover, the present study shows that within the DBS frequency range (50–200 Hz), as the stimulation frequency increases, the desynchronization speed of axon bundle accelerates at the initial phase of stimulation and the steady-state level of synchronization ratio decreases (Figure 6A). This is consistent with the observations of frequency-dependent axonal block induced by HFS in animal experiments (Jensen and Durand, 2009; Feng et al., 2013, 2014). It is well reported that the efficacy of DBS is dependent on the frequency of stimulation, with effective frequency >90 Hz (Birdno and Grill, 2008; Zhong et al., 2011; McConnell et al., 2012). The results of our simulation study suggest that at a higher stimulation frequency, DBS could replace the pathological synchronization of target neuronal populations with more asynchronous activity thereby treating the diseases. This may underline the mechanism of the frequency-dependent efficacy of DBS.

### Limitations of the Simulation Study

One of the limitations of the present study is the use of monophasic (cathodic) stimulation pulses, not biphasic pulses. Clinic DBS commonly uses asymmetrical biphasic pulses, each consisting a cathodic phase, an inter-pulse delay, and a charge-balancing anodic phase (Butson and McIntyre, 2007). The cathodic phase exerts activation effects; whereas the anodic second phase aims to balance the charge for the safety of DBS. The anodic phase is designed to have longer pulse width yet smaller amplitude in order to minimize its hyperpolarization effect, therefore it would not arrest the activation generated by the cathodic first phase. To simplify the simulation, the anodic phase was not included in the simulated pulses because of its small effects on activation. Further studies with biphasic pulses are needed to mimick the stimulations more consistent with the clinic situation.

Another limitation of the study is that the extracellular potential generated by the point source of stimulation was calculated based on homogeneous conductivity (equation 3), whereas the true extracellular volume conductor is highly heterogeneous. The heterogeneity may alter the location of largest potential change along the axon, thereby moving the position of action potential initiation to other nodes, not exactly the central node (i.e., Node_11_ in our simulation). However, no matter which node is first activated, the subsequent propagation of action potential and the reaction inside the axon would be similar because the high impedance of myelin sheath would prevent the extracellular stimulation from acting on the structures under the myelin sheath (Figure 1). It was the reaction of underneath structures (e.g., potassium accumulation) that determined the generation of intermittent block. Therefore, similar conclusions would be obtained with heterogeneous extracellular volume conductor. Nevertheless, a more realistic model accounting for heterogeneity may improve the fidelity of the model predictions.

### Summary

The present study shows that the accumulation of potassium ions in the peri-axon space during high HFS can induce intermittent block of axons, which causes asynchronous firing of action potential in an axon bundle. This desynchronized firing of axons could presumably generate asynchronous activity in the projected neurons downstream, thereby suppressing the pathological synchronization of target nuclei. The results provide important insights into the therapeutic mechanisms of DBS, which may lead to the development of novel DBS strategies and the extension of the DBS applications.

## Author Contributions

ZF and ZG designed the study. ZG, YW, and XW contributed to the model modification. ZG performed the simulation. ZF and ZG interpreted the results. ZG drafted the manuscript. ZF, XW, and YW revised the manuscript critically. All authors gave final approval of the submission.

## Conflict of Interest Statement

The authors declare that the research was conducted in the absence of any commercial or financial relationships that could be construed as a potential conflict of interest.
